# Polymorphisms analysis of the *Plasmodium ovale* tryptophan-rich antigen gene (*potra*) from imported malaria cases in Henan Province

**DOI:** 10.1186/s12936-018-2261-1

**Published:** 2018-03-23

**Authors:** Ruimin Zhou, Ying Liu, Suhua Li, Yuling Zhao, Fang Huang, Chengyun Yang, Dan Qian, Deling Lu, Yan Deng, Hongwei Zhang, Bianli Xu

**Affiliations:** 1 Department of Parasite Disease Control and Prevention, Henan Province Center for Disease Control and Prevention, Zhengzhou, 450016 People’s Republic of China; 2 National Institute of Parasitic Diseases, Chinese Center for Disease Control and Prevention, Shanghai, 200025 People’s Republic of China

**Keywords:** *Plasmodium ovale curtisi*, *Plasmodium ovale wallikeri*, *Plasmodium ovale* tryptophan-rich antigen (*potra*), Amino acid unit, Subtype

## Abstract

**Background:**

*Plasmodium ovale* has two different subspecies: *P. ovale curtisi* and *P. ovale wallikeri*, which may be distinguished by the gene *potra* encoding *P. ovale* tryptophan-rich antigen. The sequence and size of *potra* gene was variable between the two *P. ovale* spp., and more fragment sizes were found compared to previous studies. Further information about the diversity of *potra* genes in these two *P. ovale* spp. will be needed.

**Methods:**

A total of 110 dried blood samples were collected from the clinical patients infected with *P. ovale*, who all returned from Africa in Henan Province in 2011–2016. The fragments of *potra* were amplified by nested PCR. The sizes and species of *potra* gene were analysed after sequencing, and the difference between the isolates were analysed with the alignment of the amino acid sequences. The phylogenetic tree was constructed by neighbour-joining to determine the genetic relationship among all the isolates. The distribution of the isolates was analysed based on the origin country.

**Results:**

Totally 67 samples infected with *P. o*. *wallikeri*, which included 8 genotypes of *potra*, while 43 samples infected with *P. o. curtisi* including 3 genotypes of *potra*. Combination with the previous studies, *P. o. wallikeri* had six sizes, 227, 245, 263, 281, 299 and 335 bp, and *P. o. curtisi* had four sizes, 299, 317, 335 and 353 bp, the fragment sizes of 299 and 335 bp were the overlaps between the two species. Six amino acid as one unit was firstly used to analyse the amino acid sequence of *potra*. Amino acid sequence alignment revealed that *potra* of *P. o. wallikeri* differed in two amino acid units, MANPIN and AITPIN, while *potra* of *P. o. curtisi* differed in amino acid units TINPIN and TITPIS. Combination with the previous studies, there were ten subtypes of *potra* exiting for *P. o. wallikeri* and four subtypes for *P. o. curtisi*. The phylogenetic tree showed that 11 isolates were divided into two clusters, *P. o. wallikeri* which was then divided into five sub-clusters, and *P. o. curtisi* which also formed two sub-clusters with their respective reference sequences. The genetic relationship of the *P. ovale* spp. mainly based on the number of the dominant amino acid repeats, the number of MANPIN, AITPIN, TINPIN or TITPIS. The genotype of the 245 bp size for *P. o. wallikeri* and that of the 299 and 317 bp size for *P. o. curtisi* were commonly exiting in Africa.

**Conclusion:**

This study further proved that more fragment sizes were found, *P. o. wallikeri* had six sizes, *P. o. curtisi* had four sizes. There were ten subtypes of *potra* exiting for *P. o. wallikeri* and four subtypes for *P. o. curtisi*. The genetic polymorphisms of *potra* provided complementary information for the gene tracing of *P. ovale* spp. in the malaria elimination era.

## Background

*Plasmodium ovale* was first described in 1922 [1], as the fourth malaria parasite of humans [2, 3]. Generally, a *P. ovale* infection is of low parasitaemia, and the morphology of the parasite is similar to *Plasmodium vivax*. Also, it frequently presents as a mixed infection with the other *Plasmodium* species [4–9]. As a result, *P. ovale* attracted less attention compared to other species, and its prevalence has apparently been underestimated. It has long been considered predominantly found in Africa and some islands of Western Pacific [10, 11], with confirmed cases occasionally found in other endemic regions [12, 13].

Currently, *P. ovale* spp. may be classified into two different subspecies by molecular genotyping: *P. o. curtisi* (classic type) and *P. o. wallikeri* (variant type) [14]. The nuclear genome sequences further confirmed that the two species were genetically different, but morphologically indistinct [15], and their duration of latency were seemly different [16]. Both species were considered to exist sympatrically in Africa and Asia, and even both parasites were infected simultaneously [17–21].

Because of the generally low parasitaemia of *P. ovale* infections, sensitive molecular methods to detect and identify the two subspecies must be used in future investigations, with polymorphic markers as a method to discriminate the different strains. Many protocols showed that the SS rRNA genes [17, 22, 23] were suitable for identification but not for genotyping. The recent study showed that the gene encoding *P. ovale* tryptophan-rich antigen (*potra*) could be used to distinguish the two *P. ovale* subspecies [14]. The sequence and size of the tryptophan-rich antigen gene was variable among the *P. ovale* subspecies (*poctra* and *powtra*) [14]. A nested PCR detection assay was exploited to discriminate the species by the size of the amplified fragments (299 or 317 bp for *poctra*; 245 bp for *powtra*), where the conserved sequences were chosen as primers for these two genes [19]. Additionally, a semi-nested PCR protocol was developed by Tanomsing et al. [24] with which the two *P. ovale* subspecies could be discriminated efficiently, and more fragment sizes were found comparing with previous studies, the 299 bp fragment was overlapping between the two subspecies. This would invalidate amplified fragment size difference, as a means of distinguishing between *P. o. curtisi* and *P. o. wallikeri*. The amplified fragment size variations resulted from differences in the number of repeated units, which suggested that a broader range of size variants might occur. In this study, more variations of *potra* gene were observed.

## Methods

### Sample collection and DNA extraction

Dried blood spots on filter paper (Whatman 3M) were collected from patients returned from Africa with *P. ovale* infection before treatment. All the patients were diagnosed by nested PCR and microscopy. All the dried blood spots were labelled with a unique identification number, air-dried and individually placed in plastic bags with desiccant and stored at − 20 °C until laboratory analysis. DNA was extracted from the dried blood spots using a QIAamp DNA mini kit (Qiagen, Germany).

### Nested PCR amplification and DNA sequencing

The fragments of *potra* was amplified with nested PCR using the primers as described previously [14, 19]. The amplified products were identified by agarose gel electrophoresis. Bidirectional sequencing was performed for the secondary *potra* PCR products using the secondary primers by Sangon Biotech Co Ltd (Shanghai, China).

### Sequencing alignments and analysis

All the genes sequences were analysed with multiple sequence alignment using the Clustal X software. HM594180–HM594183 [19], KF018430–KF018433 [24] and KX417700–KX417704 [25] from the GenBank would be as the reference sequences of *P. ovale* spp. Phylogenetic trees were constructed using the Molecular Evolutionary Genetics Analysis (MEGA) 6.06.

## Results

### Amplification of the *potra* gene of *Plasmodium ovale* spp.

**Fig. 1:**
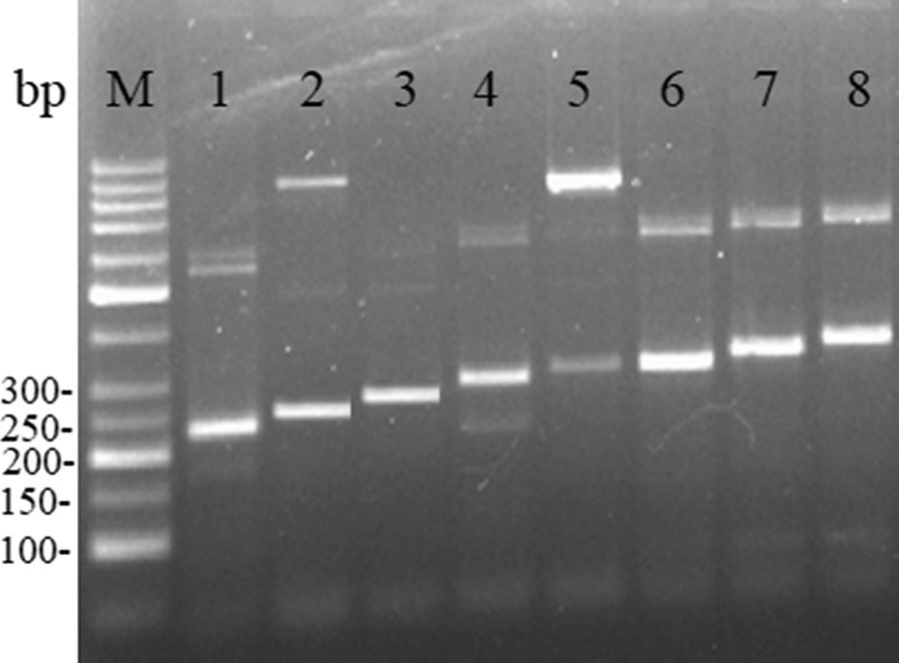
Identification of the fragment of *potra* gene by agarose gel electrophoresis. Lanes 1–5: the fragment sizes of 227, 245, 263, 281 and 299 bp for *P. ovale wallikeri*, respectively; lanes 6–8: the fragment sizes of 299, 317 and 335 bp for *P. ovale curtisi*, respectively. *M* molecular marker

A total of 110 dried blood samples, from patients returned from Africa to Henan Province with *P. ovale* infection, were collected. The amplified nested PCR products of the *potra* gene of 110 samples were blasted in the GenBank. The blast data showed that 67 samples infected with *P. o. wallikeri* and 43 samples infected with *P. o. curtisi*. More fragment sizes of the *potra* gene from this study were found comparing with the previous reports. *P. o. wallikeri* had five different sizes including 227, 245, 263, 281 and 299 bp, while *P. o. curtisi* had three sizes including 299, 317 and 335 bp. Also, the amplified fragment size differed as a result of differences in 18 bases of units, with the overlap of 299 bp between the two species (Fig. 1). The size of 245 bp was the main type for the *P. o. wallikeri*, accounting for 82.1% (55/67), and the 299 bp was the main size for *P. o. curtisi*, accounting for 67.4% (29/43). The number of isolates for each size was shown in the Table 1.

**Table 1:** Main findings of studies that sequenced the sizes of *potra* fragments

Authors	*Plasmodium ovale curtisi*	*Plasmodium ovale wallikeri*
Oguike et al. [19]	299 bp (no data)	245 bp (no data)
	317 bp (no data)	
Tanomsing et al. [24]	299 bp (n = 3)	245 bp (n = 12)
	317 bp (n = 1)	299 bp (n = 6)
	353 bp (n = 1)	335 bp (n = 2)
Zhou et al.^a^	299 bp (n = 29)	227 bp (n = 3)
	317 bp (n = 12)	245 bp (n = 55)
	335 bp (n = 2)	263 bp (n = 4)
		281 bp (n = 4)
		299 bp (n = 1)

^a^This study

Oguike reported that the sizes of the *potra* gene was 245 bp for *P. o. wallikeri* and 299 bp and 317 bp for *P. o. curtisi* [19] in Congo, Uganda and Equatorial Guinea. While there were three sizes (245, 299 and 335 bp) for *P. o. wallikeri* and three sizes (299, 317 and 353 bp) for *P. o. curtisi* in the study of Tanomsing et al. [24]. Combination with the previous studies, *P. o. wallikeri* had six sizes, 227, 245, 263, 281, 299 and 335 bp, and *P. o. curtisi* had four sizes, 299, 317, 335 and 353 bp, the fragment sizes of 299 and 335 bp were the overlaps between the two species (shown in Table 1).

### Genotypes of *potra* of *Plasmodium ovale* spp.

There were 8 genotypes of the *potra* gene for the 67 isolates infected with *P. o. wallikeri* and three genotypes of *potra* for the 43 isolates infected with *P. o. curtisi*. The sizes of 277, 245 and 263 bp for *P. o. wallikeri* all had two different subtypes. The sequences of the 11 genotypes of *potra* gene were deposited in GenBank under accession number MG588144-MG588154. For *P. o. wallikeri*, the genotype of MG588146 was the same with that of the reference sequences HM594180 and HM594181, but the reference sequences KF018430 and KF018431 were different with any of MG588144-MG588151. For *P. o. curtisi*, the genotype of MG588152 was the same with that of the reference sequences HM594182 and KF018433, and the genotype of MG588153 was the same with that of the reference sequence HM594183. Combination with the previous studies, there were ten genotypes of *potra* exited for *P. o. wallikeri*, and four genotypes for *P. o. curtisi*. The number of isolates for each genotype was shown in the Table 2.

**Table 2:** Analysis of the distinct *potra* fragments amplified from 11 genotypes of *P. ovale* and the reference sequences

Sample	*Plasmodium ovale* spp.	Size (bp)	Dominant amino acid repeats	No. of samples	GenBank accession no.
1-227	*wallikeri*	227	(MANPIN)0(AITPIN)2	1	MG588144
2-227	*wallikeri*	227	(MANPIN)1(AITPIN)1	2	MG588145
3-245	*wallikeri*	245	(MANPIN)1(AITPIN)2	1	MG588146
4-245	*wallikeri*	245	(MANPIN)2(AITPIN)1	54	MG588147
5-263	*wallikeri*	263	(MANPIN)2(AITPIN)2	3	MG588148
6-263	*wallikeri*	263	(MANPIN)1(AITPIN)3	1	MG588149
7-281	*wallikeri*	281	(MANPIN)2(AITPIN)3	4	MG588150
8-299	*wallikeri*	299	(MANPIN)2(AITPIN)4	1	MG588151
HM594180^a^	*wallikeri*	245	(MANPIN)1(AITPIN)2	–	
HM594181^a^	*wallikeri*	245	(MANPIN)1(AITPIN)2	–	
KF018430^a^	*wallikeri*	299	(MANPIN)3(AITPIN)3	–	
KF018431^a^	*wallikeri*	335	(MANPIN)5(AITPIN)3	–	
9-299	*curtisi*	299	(TINPIN)3(TITPIS)1	29	MG588152
10-317	*curtisi*	317	(TINPIN)3(TITPIS)2	12	MG588153
11-335	*curtisi*	335	(TINPIN)4(TITPIS)2	2	MG588154
HM594182^a^	*curtisi*	299	(TINPIN)3(TITPIS)1	–	
HM594183^a^	*curtisi*	317	(TINPIN)3(TITPIS)2	–	
KF018432^a^	*curtisi*	353	(TINPIN)6(TITPIS)1	–	
KF018433^a^	*curtisi*	299	(TINPIN)3(TITPIS)1	–	

^a^GenBank accession no.; –: no date

### Alignment of the translated amino acid sequence of *potra* fragments

**Fig. 2:**
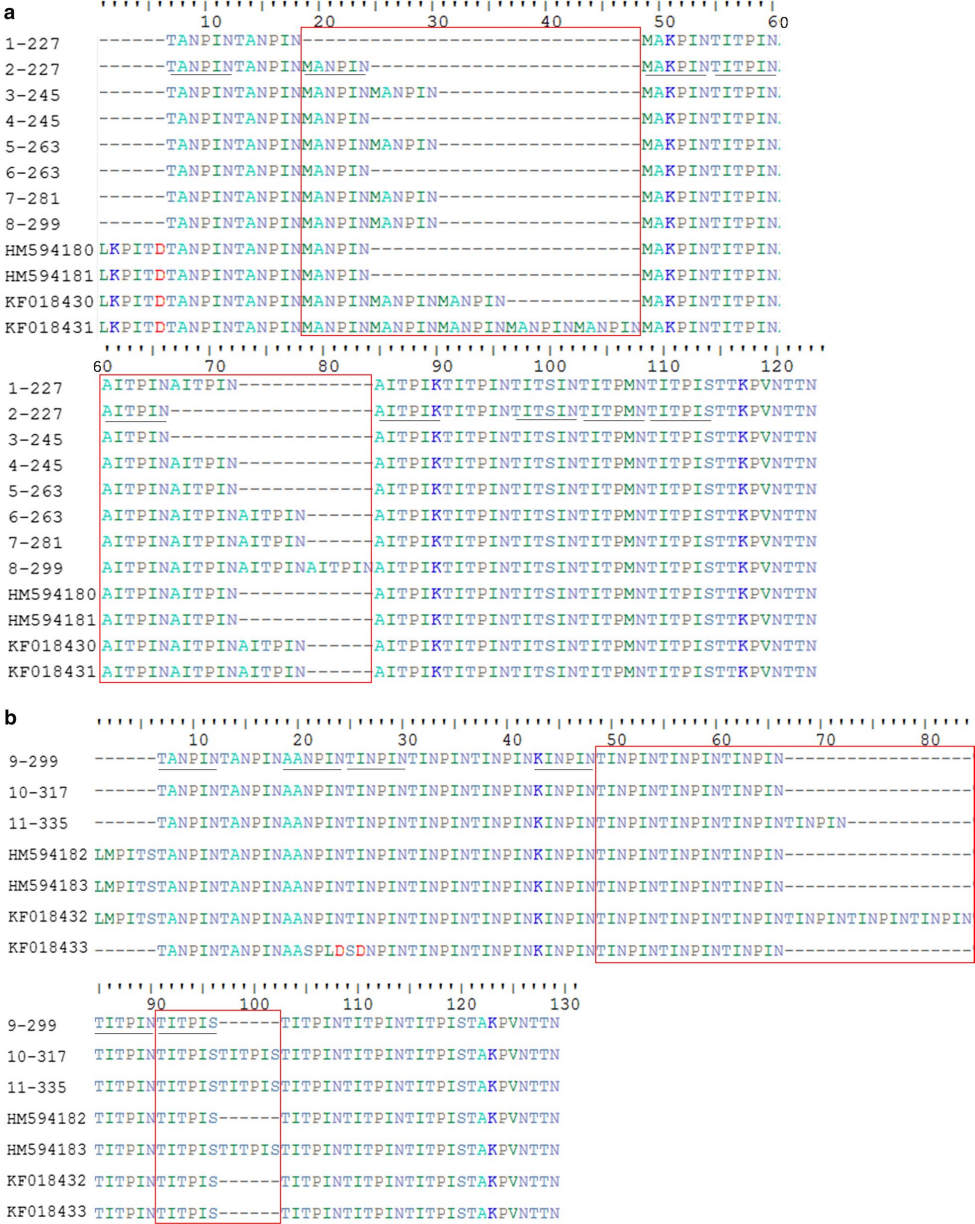
Amino acid sequence alignment of the distinct *potra* fragments amplified from 11 genotypes of *Plasmodium ovale* and the reference sequences. **a** *P. ovale wallikeri*; **b** *P. ovale curtisi*. Six amino acids were one amino acid unit which were underlined. *P. o. wallikeri* was mainly composed with nine kinds of amino acid units, TANPIN, MANPIN, MAKPIN, TITPIN, AITPIN, AITPIK, TITSIN, TITPMN and TITPIS. While *P. o. curtisi* had six kinds of amino acid units, TANPIN, AANPIN, TINPIN, KINPIN, TITPIN and TITPIS. Boxed sequences represented the dominant amino acid repeats that differed in the number of amino acid units. The sequences of *P. o. wallikeri* were characterized by two amino acid units, MANPIN and AITPIN. The sequences of *P. o. curtisi* were characterized by amino acid units TINPIN and TITPIS

Interestingly, the translated amino acid sequence of *potra* fragments were composed with multiple amino acid units, and six amino acids was considered as a unit. For *P. o. wallikeri*, it was mainly composed with nine kinds of amino acid units, TANPIN, MANPIN, MAKPIN, TITPIN, AITPIN, AITPIK, TITSIN, TITPMN and TITPIS. While *P. o. curtisi* had six kinds of amino acid units, TANPIN, AANPIN, TINPIN, KINPIN, TITPIN and TITPIS. The two subspecies of *P. ovale* had three common amino acid units, TANPIN, TITPIN and TITPIS. Amino acid sequence alignment revealed that the types of *P. o. wallikeri* differed in two amino acid units, MANPIN and AITPIN, while the types of *P. o. curtisi* differed in amino acid units TINPIN and TITPIS. The amplified fragment size differed as a result of differences in amino acid of repeat units. The dominant amino acid repeats of *potra* were showed in Table 2. As the same with the genotypes of *potra*, there were 10 subtypes exiting for *P. o. wallikeri* and 4 subtypes for *P. o. curtisi*. The detail information is shown in Fig. 2 and Table 2.

### Phylogenetic relationship among *potra subtype families*

**Fig. 3:**
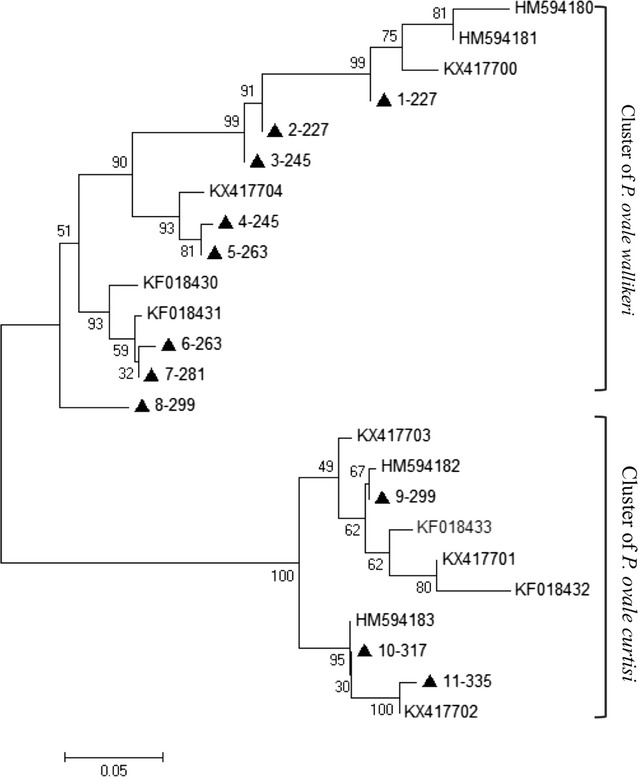
Genetic relationship of *potra* among isolates of *Plasmodium ovale* subspecies. Black triangles represented the isolates in this study. The reference sequences of *P. ovale* spp., HM594180–HM594183, KF018430–KF018433 and KX417700–KX417704, were obtained from the NCBI database

Neighbour-joining was used to cluster the *potra* gene sequences. The 11 genotypes were classified into two clusters, eight genotypes infected with *P. o. wallikeri* were as one cluster and other three genotypes infected with *P. o. curtisi* formed the other one. Meanwhile the cluster of *P. o. wallikeri* was classified into five sub-clusters, distinctive phylogenetic relationship presented between the eight genotypes of *P. o. wallikeri*. The sequence 1-227, formed a sub-cluster with reference sequences HM594180, HM594181 and KX417700, having the same 2 repeats of AITPIN. The sequences 2-227 and 3-245 existed in the same sub-cluster with a closer genetic relationship, having the same repeat of MANPIN. The sequences 4-245 and 5-263 formed a sub-cluster with reference sequence KX417704, having the same two repeats of MANPIN. The sequences 6-263 and 7-281 formed another sub-cluster with reference sequences KF018430 and KF018431, having the same three repeats of AITPIN. Having significantly difference sequences, the three genotypes of *P. o. curtisi* also formed two sub-clusters. The sequence 9-299 formed a sub-cluster with reference sequences, having the same repeat of TITPIS. The sequences 10-317 and 11-335 had a closer genetic relationship, which formed another sub-cluster with reference sequences, having the same two repeats of TITPIS (Fig. 3).

### Distribution of the *Plasmodium ovale* spp.

All the 110 *P. ovale* patients returned from twenty counties of Africa, and 70.0% (77/110) were from Angola, Congo, Equatorial Guinea, Liberia and Nigeria. Only one patient originated from Algeria, Tanzania, Sudan, Libya and Ivory Coast. 82.1% (55/67) *P. o. wallikeri* infection were the same subtype with 245 bp size of the *potra* gene, and this type was identified in 16 countries except Gabon, Algeria, Tanzania and Libya; three isolates (4.5%, 3/67) with 227 bp size originated from Congo and Liberia,; four isolates (6.0%, 4/67) of 263 bp size were from Angola, Liberia and Gabon; 4 isolates (6.0%, 4/67) of 281 bp size were from Congo, Liberia, Nigeria and Sierra Leone; only one isolate of 299 bp size was from Congo. In contrast, the predominant type of *P. o. curtisi* (67.4%, 29/43) was the *potra* gene with 299 bp size, which originated from 12 countries; 27.9% (12/43) with 317 bp size and 4.7% (2/43) with 335 bp size were also identified from nine countries and two countries, respectively (Table 3).

**Table 3:** Distribution of the *P. ovale* spp. based on the different size for the isolates

Origin conutry	*Plasmodium ovale wallikeri*						*Plasmodium ovale curtisi*				Total
	227 bp	245 bp	263 bp	281 bp	299 bp	Subtotal	299 bp	317 bp	335 bp	Subtotal	
Angola	0	12	2	0	0	14	5	2	0	7	21
Congo	2	7	0	1	1	11	2	1	1	4	15
Equatorial Guinea	0	8	0	0	0	8	5	1	0	6	14
Liberia	1	7	1	1	0	10	4	1	0	5	15
Nigeria	0	4	0	1	0	5	4	3	0	7	12
Ghana	0	1	0	0	0	1	3	1	0	4	5
Cameroon	0	3	0	0	0	3	0	0	1	1	4
The Republic of Guinea	0	2	0	0	0	2	0	1	0	1	3
Uganda	0	3	0	0	0	3	0	0	0	0	3
Sierra Leone	0	1	0	1	0	2	1	0	0	1	3
Benin	0	2	0	0	0	2	0	0	0	0	2
Chad	0	1	0	0	0	1	0	1	0	1	2
Mozambique	0	1	0	0	0	1	1	0	0	1	2
Zambia	0	1	0	0	0	1	1	0	0	1	2
Gabon	0	0	1	0	0	1	1	0	0	1	2
Algeria	0	0	0	0	0	0	0	1	0	1	1
Tanzania	0	0	0	0	0	0	1	0	0	1	1
Sudan	0	1	0	0	0	1	0	0	0	0	1
Libya	0	0	0	0	0	0	1	0	0	1	1
Ivory Coas	0	1	0	0	0	1	0	0	0	0	1
Total	3	55	4	4	1	67	29	12	2	43	110

Two genotypes for the 227 bp size, MG588144 and MG588145, just one isolate of MG588144 genotype returned from Congo

Two genotypes for the 245 bp size, MG588146 and MG588147, just one isolate of MG588146 genotype returned from Liberia

Two genotypes for the 263 bp size, MG588148 and MG588149, just one isolate of MG588149 genotype returned from Liberia

## Discussion

Malaria elimination is a long-term goal to be achieved worldwide. As one species of human Plasmodium, the identification of *P. ovale* is more widespread than formerly known. *Plasmodium ovale,* like *P. vivax,* has hypnozoites that cause relapses [26, 27], and it consists of two different subspecies: *P. ovale curtisi* and *P. ovale wallikeri* [14]. Therefore, the differentiation of the two *P. ovale* species, especially with respect of molecular phylogeny will need to be better understood.

In 2011, Oguike et al. [19] published the discrimination of the two *P. ovale* subspecies by the size of the amplified fragments of the *potra* gene (299 or 317 bp for *P. o. curtisi*; 245 bp for *P. o. wallikeri*), using nested PCR. Although this technique was specific for *P. ovale* spp., the sizes of the amplified fragment varied with the number of repeat units, which reduced the discrimination between species: for *P. o. curtisi*, 299, 317 and 353 bp, and for *P. o. wallikeri*, 245, 299 and 335 bp [24]. Tanomsing et al. [24] suggested the number of *potra* size variations might be more than those evaluated and this speculation was confirmed in this study. Using the same primes and method, more fragment sizes were identified in this study, while some sizes overlapped between the two subspecies: for *P. o. curtisi* having sizes 299, 317 and 335 bp, and for *P. o. wallikeri* having sizes 227, 245, 263, 281 and 299 bp. The results of the three studies [19, 24] were also combined, as shown in Table 1. Four different sizes for *P. o. curtisi* and six sizes for *P. o. wallikeri* have been reported, and that the fragment sizes of 299 and 335 bp were overlaps between the two species. As more samples were analysed, it was likely that the number of *potra* size variants would be more than expected. Conceivably, more size variants may be identified in future studies.

*Potra* gene was used to discriminate the two *P. ovale* species, because the tryptophan-rich antigen was encoded by a repeat pattern of variable length 3-amino acid [14]. Sutherland et al. [14] and Oguike et al. [19] had also proposed that the *potra* gene of *P. o. curtisi* (*poctra*) could be identified by the pattern of the six amino acids of the repeat region, TITPIS, while the *potra* gene of *P. o. wallikeri* (*powtra*) were different in two non-synonymous positions. By alignment of the amino acid sequence of *potra* fragments, 11 genotypes of *potra* were found from the 110 isolates in this study. Combination with the previous studies of Oguike et al. [19] and Tanomsing et al. [24], showed ten subtypes of *potra* gene for *P. o. wallikeri* and four subtypes for *P. o. curtisi*, and 14 genotypes of *potra* gene were under analysis. This study showed that the amino acid sequence of *potra* fragments were composed with multiple amino acid units, six amino acids were as one unit. There was different dominant amino acid repeat of *potra* for the two *P. ovale* species, which could be used to discriminate the subtype of *P. ovale* spp. The repeat of six amino acids as one unit to analyse the difference of the genotypes between the two *P. ovale* species was first reported, which could make the results more clear and simple. The sizes of 277, 245 and 263 bp for *P. o. wallikeri* all had two different subtypes, and this phenomenon did not find in the *P. o. curtisi*.

The sizes of the reference sequences KX417700–KX417704 [25] from Genbank were short for discriminating the differences of dominant amino acid repeats. In our study, the genetic relationship of the *P. ovale* spp. was analysed by the neighbour-joining tree mainly based on the number of the dominant amino acid repeats including MANPIN, AITPIN, TINPIN or TITPIS.

The distribution of the two *P. ovale* spp. was different. The isolates of *P. o. wallikeri* was more than that of *P. o. curtisi*, and the genotype of the 245 bp size was the predominant type for *P. o. wallikeri* in most Africa countries, but the other genotypes were less. For *P. o. curtisi*, the genotypes of the 299 and 317 bp size were commonly in Africa and the genotype of 335 bp size was less.

Molecular epidemiological studies on genetic diversity of *Plasmodium vivax* have been based mainly on single copy polymorphic genes which code for parasite surface antigens such as circumsporozoite protein (*csp*), merozoite surface protein-1 (*msp*-*1*) and merozoite surface protein 3 alpha (*msp 3α*) [28]. *Pvcsp* comprises of central domain of tandem repeated sequences flanked by two non-repeated conserved sequences [29–32]. Two types of repeat elements, either VK210 or VK247 types were detected in clinical isolates of *P. vivax* and thus *pv*csp serves as a useful tool for genotyping [33, 34]. *Potra* has the similar characteristics with *pvcsp*, which also could be used for parasite genotyping.

## Conclusions

Considering the change of malaria epidemiology and the approaching of malaria elimination, *P. ovale* spp. deserves more attention. Molecular techniques are a good tool for detecting and identifying the two *P. ovale* subspecies and their relative distribution.

## Abbreviations

*Potra*: *Plasmodium ovale* tryptophan- rich antigen
Spp.: plural form of species
PCR: polymerase chain reaction
NCBI: national center for biotechnology information
DNA: desoxyribonucleic acid
MEGA: molecular evolutionary genetics analysis
